# Are There Postnatal Benefits to Prenatal Kick Counting? A Quasi-Experimental Longitudinal Study

**DOI:** 10.3389/fpsyg.2022.712562

**Published:** 2022-01-26

**Authors:** Brenna Owens, Klaus Libertus

**Affiliations:** Department of Psychology, University of Pittsburgh, Pittsburgh, PA, United States

**Keywords:** maternal depression and anxiety, fetal kicks, motor development, parent-child engagement, remote observation

## Abstract

Mild signs of postpartum depression or anxiety are present in up to half of all new mothers. However, the impact of having the “baby blues” on infant development remains largely unknown. The current study explores a potential relation between mother’s self-reported depression or anxiety symptoms and infant’s motor development in a longitudinal sample of 50 mother-infant dyads. Further, we examine whether engaging in fetal kick counting during pregnancy may reduce maternal psychopathology symptoms and thereby positively influence infant motor development and parent-child engagement during the first months of life. We hypothesized that subclinical maternal psychopathology would negatively impact infant motor development, and that completing a fetal kick count activity during the third trimester would reduce overall signs of maternal psychopathology. Results only partially support these hypotheses. Postpartum maternal anxiety seems to negatively affect the emergence of infants’ fine motor skills. However, engaging in fetal kick counting during pregnancy did not reduce maternal depression or anxiety symptoms. Nevertheless, preliminary evidence suggests that engaging in fetal kick counting may impact early child development by altering the mother’s attitudes toward the child. Future research is needed to examine the value of this low-cost intervention strategy more closely.

## Introduction

According to estimates reported by the CDC (Bauman et al., 2020), about one in eight mothers (12.5%) may experience depression or anxiety symptoms shortly after childbirth. Experiences of postpartum depression or anxiety can turn what should be one of the happiest times in a woman’s life into a period of dread and despair. The period following birth is crucial for mother-infant bonding and attachment development (Ainsworth, 1979; Chess and Thomas, 1982). Consequently, postpartum maternal psychopathology may negatively impact the emerging bond between the mother-child dyad. Indeed, studies examining the impact of maternal psychopathology in the context of *clinical* levels of depression or anxiety support this view by reporting negative impacts of maternal psychopathology on children’s development (e.g., Cicchetti et al., 2000; Milgrom et al., 2004; Cornish et al., 2005; Castelli et al., 2015). However, less is known about the impact of *subclinical* psychopathology on child development. Subclinical psychopathology is commonly referred to as the “baby blues” and has and has a higher estimated prevalence of around 40 to 50% in new mothers (Rezaie-Keikhaie et al., 2020). Symptoms related to the “baby blues” do not meet threshold for a clinical diagnosis of postpartum depression or anxiety, but nevertheless may have a similar impact on early child development. Further, untreated subclinical symptoms may eventually spiral into more sever psychopathology (Fogel et al., 2006). The high prevalence of clinical or subclinical postpartum psychopathology, combined with its potential negative consequences for both the mother and child development, make this issue a serious public health challenge. Therefore, the current study examines the impact of mild maternal psychopathology on infants’ early motor development and whether a simple mother-fetus bonding activity (fetal kick counting; FKC) may reduce levels of maternal psychopathology.

### Impact of Maternal Psychopathology on Infant Development

Several studies have documented negative effects of clinical maternal depression or anxiety on child development (e.g., Cummings and Davies, 1994; Bernard-Bonnin et al., 2004). Results of these studies suggest that maternal depression or anxiety can negatively impact a child’s language development (Brookman et al., 2020), motor development (Piallini et al., 2016), attachment (Lindgren, 2001), emotional development (Ibanez et al., 2015), or school readiness skills (Wang and Dix, 2013). Specifically, infants of depressed mothers have been reported to be less vocal and score lower on standardized language assessments at 18 months (Brookman et al., 2020). Others report that maternal depression may impact an infant’s language development as early as 9 months of age (Paulson et al., 2009). Similar findings exist regarding infants’ psychomotor development (Cornish et al., 2005) and their social-emotional development (Fredriksen et al., 2019). Finally, long-term consequences of maternal depression and anxiety during infancy have been noted to include lower verbal and full-scale IQ scores at three to 4 years of age (Cicchetti et al., 2000; Milgrom et al., 2004). Together, these findings show that maternal psychopathology can impact a child’s early development across domains and may have long-lasting impacts on developmental outcomes.

However, there have been some inconsistencies in the literature regarding the impact of maternal psychopathology—especially in the domain of motor development. For instance, Piallini et al. (2016) assessed the influence of subclinical and normative maternal depressive symptoms on infant motor development and report that children of mothers exhibiting higher levels of depression symptoms seem to score overall *better* on standardized motor assessments. In contrast, another study assessing the influence of postpartum depression on motor development found that infants with chronically depressed mothers scored significantly *lower* on motor assessments, including delayed walking onset at 15 months of age (Cornish et al., 2005). These conflicting findings suggest that the kind and severity of symptoms may determine whether maternal psychopathology can have a negative or positive impact on early child development. This is an important observation highlighting the need for further research on the unique impact of maternal depression and anxiety symptoms on child development (O’Connor et al., 2002; Ibanez et al., 2015; Schmidt et al., 2016; Brookman et al., 2020). Maternal anxiety and depression show high co-occurrence and are closely associated, but findings suggest that maternal depression has a stronger impact on child development than anxiety (Barker et al., 2011). Whether there are differences in how anxiety and depression influences child development remains unknown.

### Maternal Psychopathology and Developmental Cascades

The long-lasting and domain crossing impact of maternal psychopathology suggests the initiation of a developmental cascade (Masten and Cicchetti, 2010). One potential mechanism underlying this developmental cascade could be that maternal psychopathology has a negative effect on mother-infant interactions. Mother-infant interactions are critical for early child development by providing children with important experiences and learning opportunities. If maternal psychopathology disrupts subsequent mother-infant interactions, then this would consequently also impact infant development in other domains. Specific details about the mother-child dyad, such as the infant’s gender, may further moderate the impact of postpartum depression throughout different developmental stages. For example, studies suggest that boys may be more vulnerable to the influence of maternal psychopathology, especially during infancy (Murray et al., 1993; Katherine Weinberg et al., 2006). However, others have found both genders to be impacted similarly by maternal psychopathology (Cornish et al., 2005). These findings suggest that child gender should be considered when examining the impact of maternal depression or anxiety. Further, maternal psychopathology seems to initiate a developmental cascade that impacts subsequent child outcomes—potentially by impacting the quality of early mother-infant interactions.

### Mother-Infant Interactions

Maternal responsiveness and sensitivity to their child during mother-infant interactions play a pivotal role in early child development (Cicchetti et al., 2000). Both mother and infant mutually contribute to the quality of interactions through the use of responsiveness to verbal cues, infant eye-gaze, and maternal touch (Van Egeren et al., 2001). Unfortunately, research suggests that maternal depression and anxiety symptoms can negatively impact the quality of parent-infant interactions (Paulson et al., 2009). For example, mothers reporting postpartum depressive symptoms also experience impaired mother-infant bonding and report more negative perceptions regarding their child’s behavior and abilities (Tronick and Reck, 2009). Potential reasons for the decline in parent-infant interaction quality may be that mothers experiencing depressive symptoms have been shown to exhibit negative characteristics such as being more intrusive, withdrawn, and hostile, creating a negative environment to facilitate child development (Wang and Dix, 2013). Further, mothers experiencing postpartum depression are significantly less responsive to their infant (Milgrom et al., 2004) and engage in less conversational turn-taking (Brookman et al., 2020). Together, these results demonstrate that maternal depression and anxiety may indeed have negative influences on mother-child interactions during the first years of life.

### Prenatal Influences

Conditions such as postpartum depression or the “baby blues” are commonly considered to affect new mothers following birth. However, maternal psychopathology may occur already during pregnancy. Prenatal maternal psychopathology can negatively influence child development prior to the child’s birth (Buist, 2002). For example, mothers who experienced depressive symptoms during pregnancy have been found to engage in fewer positive health practices such as taking prenatal vitamins, visiting obstetric care, or engaging in exercising, while simultaneously being more likely to engage in more negative health practices such as drug or alcohol usage (Lindgren, 2001). Such behavior patterns may increase the likelihood of negative influences on fetal development stemming from poor nutrition or teratogens. Others investigated the relation between ruminative thinking and depressed mood during pregnancy, postpartum depression, and mother-infant bonding. Results reveal that depressive symptoms during the prenatal period predict subsequent postpartum depression symptoms poor mother-child bonding (Müller et al., 2013).

Subclinical maternal psychopathology symptoms are highly prevalent during pregnancy and may negatively impact maternal-fetal attachment later in pregnancy (Lindgren, 2001). Therefore, this period may offer a unique opportunity for intervention before symptoms worsen or continue (Buist, 2002). Before mothers even meet their child and begin their new chapter together, maternal psychopathology may already have a lasting impact on the mother-child relationship and subsequent child development. Therefore, identifying practices that can help ameliorate maternal psychopathology symptoms during pregnancy could have far reaching positive effects on child development.

### Risk Factors for Maternal Psychopathology

Many variables have been identified as potential risk factors for postpartum depression or anxiety. Identified risk factors include the presence of depression or anxiety during pregnancy (Davey et al., 2011), maternal age (Matsumoto et al., 2011), lack of social support (Robertson et al., 2004) and major life events (Milgrom et al., 2008). When investigating the prevalence of postpartum depression in various age groups, it was found that rates of postpartum are highest in the youngest (< 25) and oldest (35 +) mothers (Matsumoto et al., 2011). It was theorized that older mothers (35 +) may experience postpartum depression due to maladjustment to their novel roles as mothers both in aspects of social/cognitive and attachment adjustment (Carolan, 2005). Younger mothers on the other hand may not be as developmentally prepared in comparison to their older counterparts, as emerging adult mothers have been shown to exhibit less positive regard, supportiveness, and sensitivity in addition to exhibiting more negative regard and intrusiveness (Lewin et al., 2013).

While it is not possible to prevent every possible risk factor during the postpartum period, one can engage in preventative activities. However, to engage in preventative measures, it is first necessary to identify activities that may be beneficial. Only few studies have examined this important issue. For example, it has been reported that attachment-facilitating activities such as breastfeeding can be beneficial to mothers and combat postpartum depression (Figueiredo et al., 2014). This observation suggests that other attachment-facilitating activities may also be beneficial to mothers during the prenatal period. Indeed, breastfeeding is a simple and effective attachment-facilitating activity for mothers and their infants to engage in postpartum. However, ideally expectant mothers would engage in attachment-facilitating activities already *during* pregnancy to begin the process of mother-infant attachment building early on. Such a “preemptive” approach may avoid subsequent issues and may be easier to implement for the mother. Therefore, there is a need to explore potential prenatal intervention activities that may reduce maternal psychopathology and improve mother-infant attachment and engagement.

### Fetal Kick Counting

The prenatal period offers a relatively underutilized window of opportunity to prevent the emergence of maternal psychopathology. While there may be pharmacological interventions available, a cheaper and less intrusive intervention may be engaging in fetal kick counting (FKC). Fetal kick counting asks mothers to take a moment each day and be attentive to their fetal movements. One medical use of FKC is to check on fetal wellbeing by quantifying the movements. Therefore, FKC is used in high-risk pregnancies, allowing them to detect any abnormal or decreased movement patterns and alert their physician, preventing from possible stillbirth (Lobb et al., 1985). In addition, FKC may also benefit maternal mental health and encourage early mother-child bonding but findings on this emerging intervention strategy are still mixed. Some studies report no effect of engaging in FKC on maternal mental health (Liston et al., 1994; Delaram et al., 2017), while others have found that mothers engaging in FKC to report enhancement on their general health status, social functioning, anxiety and depressive symptoms, and insomnia (Saastad et al., 2012; El-Sayed et al., 2018). One theory regarding why FKC may benefit maternal wellbeing is attending to fetal movements facilitates maternal-fetal attachment (Saastad et al., 2011). These inconclusive findings show that more research on the potential impact of FKC on maternal mental health is needed.

### The Current Study

The current study provides a preliminary exploration of the relation between subclinical maternal psychopathology symptoms on infant motor development during the first months of life. Further, we explore whether engaging in FKC during the last trimester of pregnancy may reduce the emergence of subclinical maternal psychopathology symptoms such as negative perceptions toward their child. We hypothesize that already the presence of *subclinical* postpartum psychopathology (i.e., anxiety and depression) will have a negative impact on infants’ early motor development and may also increase parent’s negative views regarding their child’s development. Further, we predict that engaging in a 2-week FKC activity during pregnancy will reduce postpartum psychopathology symptoms and mother’s endorsement of negative perceptions about their child. Finally, we will explore whether fetal activity levels are related to infant’s subsequent motor development, and whether engaging in FKC during pregnancy enhances the quality of subsequent mother-infant engagement quality during play. We will examine these questions with a fully remote sample of mother-child dyads, completing all assessments *via* online surveys and live video conferencing.

## Materials and Methods

All study procedures and measures were approved by the University’s Internal Review Board. Participants completed informed consent before engaging in any study procedures. Mothers completed consent on behalf of themselves and of their child.

### Participants

Participants were recruited *via* posts on social media and digital ads in local obstetrician-gynecologist and pediatrician offices for an ongoing longitudinal study examining the factors influencing early motor development. Participants included a total of 50 mother-child dyads divided into two groups based on the time of their entry into the study. One group of mother-child dyads enrolled in the study during the third trimester of pregnancy (after 28 weeks gestation) and completed a daily kick-count survey for a period of 2 weeks (“Kick Count Group” or K+; *n* = 25; 11 female). All mothers in this group were asked to complete the kick-counting activity around 30 to 34 weeks of gestation. A second group of mother-child dyads enrolled into the study following their child’s birth and consequently did not complete the kick-count survey (“No Kick Group” or K−; *n* = 25; 12 female). Mothers in this group joined the study between 1- to 3-months following the birth of their child. It is important to note that *group membership is not random in our design*: Group membership was determined by the timing of enrollment into the study (during vs. after pregnancy). However, families in both groups enrolled into a larger longitudinal study including repeated parent-child observations over the next 24 months. Further, demographics variables show no differences between the K+ and K− groups (see Table 1). Therefore, it seems unlikely that systematic differences between the two groups existed due to the timing of enrollment.

**TABLE 1:** Participant demographic information.

	K− group	K+group	*t*-test comparison
*N*	25	25	—
Parent age (years)	32.04 (4.71)	32.36 (2.77)	*p* = 0.771
Parent education (7-point scale)	4.19 (1.49)	4.73 (0.75)	*p* = 0.179
Family income (12-point scale)	9.47 (3.06)	9.80 (3.02)	*p* = 0.745
Child age (days)	118.70 (25.20)	113.13 (23.18)	*p* = 0.499
Child Gestational Age (weeks)	39.20 (1.80)	39.38 (0.60)	*p* = 0.647
Child weight at birth (grams)	3197.71 (639.60)	3503.40 (392.40)	*p* = 0.075
Child Race	12 White 5 POC 8 unknown	17 White 4 POC 4 unknown	—

*Values in parentheses are standard deviations. Parent education was scored on a 7-point scale, with higher numbers indicating more years of education (ranging from no high school degree to doctorate). Family income was scored on a 12-point scale, with higher numbers indicating higher income levels (range less than 10 k to more than 150 k). POC stands for “Person of Color” and includes Black, Asian, and mixed race.*

### Measures

#### Fetal Movements

Mother-child dyads in the K+ group (enrolled during pregnancy) were asked to complete a daily kick-count survey for a period of 14 days during their third trimester of pregnancy. To complete the survey, mothers were asked to identify a time during their day that is convenient for them, sit or lie down, place their hands on their stomach, and pay attention to their child’s movements for a duration of 10 min. Mothers were instructed to count each felt movement of their child that is separated by at least 2 s of inactivity as one kick. An online diary was used to collect the daily kick counts and allowed mothers to enter the actual duration of their observation. Using this information, fetal movements rate per minute was calculated for each mother-child dyad. Emails and notes within the diary were used to remind parents to complete the daily sessions. While mothers were encouraged to complete the kick-counting diary around the same time each day, this was not monitored.

#### Maternal Psychopathology

Symptoms of postnatal depression, anxiety, or other psychopathology were assessed for mothers in both the K+ and K− group during the first 6 months following the birth of their child using the Symptom Checklist 90-Revised (SCL90-R; Derogatis, 1983). Mothers completed the SCL-90R at home using an online version of the measure between 1- to 4-months following the birth of their child. The SCL-90 is a self-report questionnaire consisting of 90 items designed to capture a range of psychopathology symptoms. The instrument yields three global indices and nine primary symptom dimensions. Individual symptom dimensions are somatization, obsessiveness-compulsiveness, interpersonal sensitivity, depression, anxiety, hostility, phobic anxiety, paranoid ideation, and psychoticism. However, the current study focuses only on the depression and anxiety-related dimensions (i.e., anxiety, phobic anxiety, and paranoid ideation), as these have been reported to be most prevalent during the postpartum period (Reck et al., 2008). For analysis, the three anxiety related dimensions were compounded into one comprehensive anxiety score. SCL-90 items are rated on a 5-point scale ranging from “Not at all” (0) to “Extremely” (4). Scores across 13 depression related items and 23 anxiety related items were averaged, resulting in final scores ranging from 0 to 4 for the depression and anxiety dimensions, respectively. For the current sample of mothers, mean depression ratings (*M* = 0.56, *SD* = 0.55) and mean anxiety ratings (*M* = 0.24, *SD* = 0.36) were low confirming that our participants do not show signs of clinical depression or anxiety.

#### Infant Motor Development

Infant motor development was assessed in both the K+ and K− group at 3 months of age using the Early Motor Questionnaire-Extended (EMQ-X) when the infant was around 4 months of age (*M* = 3.83 months, *SD* = 0.79). The EMQ-X is a slight extension of the EMQ, a parent-report measure of early motor skills that has been validated for accuracy in the past (Libertus and Landa, 2013). However, for the age-range tested in the current study, the EMQ and EMQ-X are identical. The EMQ-X includes 140-item parent-report measure that assesses infants’ gross motor (GM, 50 items), fine motor (FM, 50 items), and perception-action skills (PA, 40 items). Parents completed an online version of the EMQ-X from their own homes. The original EMQ correlates highly the Mullen Scales of Early Learning (Mullen, 1995) and the Peabody Developmental Motor Scales (Folio and Fewell, 2000), two gold-standard measures of early motor development (Libertus and Landa, 2013).

#### Parent Perceptions

In addition to assessing infants’ motor skills, the EMQ-X also introduces a 20-item parent perceptions scale. This new measure asks parents how they feel about parenting in general (10 items) and their child in particular (10 items). This measure was designed around four conceptual sub-scales intended to capture parents’ positive perceptions toward being a parent, negative perceptions toward being a parent, positive perceptions toward their child, negative perceptions toward their child. However, this questionnaire has not been validated to-date and its use should be considered exploratory. For full dissemination and future adoption, the full parent perceptions scale is provided in Supplementary Appendix 1.

#### Maternal Engagement

Maternal engagement was examined in a longitudinal follow-up when the child was around 3 months of age. Mother-child dyads were observed during a 5-min free play session in their own home. Visits were completed virtually *via* video conference and mothers were instructed to play with their child as they “normally would.” This approach increases ecological validity and reduces distractions of the dyad during the interaction and has been used in previous research (Libertus and Violi, 2016). Video recordings of the interactions were captured remotely and subsequently coded by trained observers for maternal engagement using the coding scheme outlined in Table 2. Video records were coded in 1-minute-long intervals, resulting in a total of five codes for each mother-child dyad. For analysis, the average engagement score across all 5 min of play was calculated for each participant. Coders were blind to participant’s group membership. Please note that the coding scheme was developed for the current study and has not been validated or tested in prior research.

**TABLE 2:** Maternal engagement coding scale.

Scale	Engagement criteria
0	Child alone in room, parent not visible or audible.
1	Parent is present but not talking to child.
2	Parent is talking and looking at the child, but only provides directives and supportive words (e.g., “go here,” “thank you”).
3	Parent is talking and looking at the child, but exchange is one-sided and limited to parental elaboration (descriptions), conversation (questions), or positive praise (e.g., “it’s a car!,” “good job,” “what do you think?”).
4	Same as level 3, but exchange between parent and child is interactive and includes turn-taking or sharing. During the exchange, parent and child are looking at each other (i.e., face-to-face interaction). Parent and child respond to any bids made by the other partner (e.g., Child touches a block and parent states “it’s a block”).

*Engagement was coded in 1-min segments. The highest code observed within the segment was coded. If an exchange started within 1 min and continued into the next, the scale level was assigned to both segments (if this was the highest observed level).*

### Procedure

All participants were invited to participate in this research study *via* email and received an online consent form. Following consent completion, families received all study-related surveys and materials *via* email. Families were first asked to complete a demographic questionnaire, asking about their child’s gender, race, ethnicity, family education levels, and family income. Next, families in the K+ group received the fetal movements survey during their third trimester of pregnancy. Following birth of their child, mothers in both the K+ and K− groups received the SCL-90R questionnaire to assess maternal psychopathology. Finally, when their child was around 4 months of age, families in both the K+ and K− groups were asked to complete the EMQ.

## Results

### Relation Between Postpartum Maternal Psychopathology and Infant Motor Development

**FIGURE 1:**
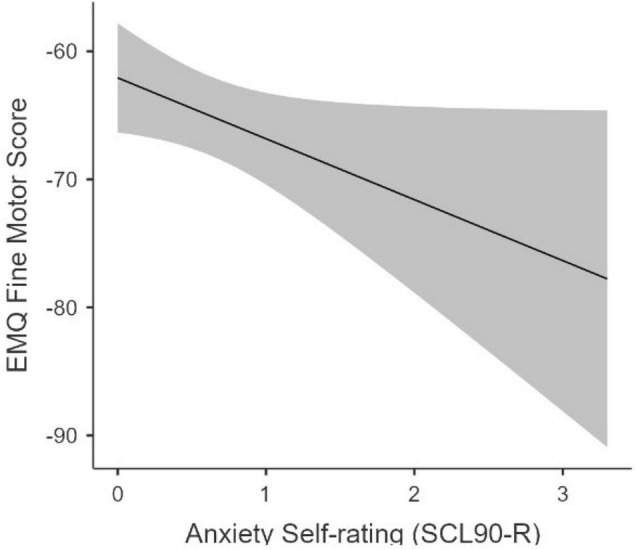
Relation betweenMaternal Anxiety and Infant Fine Motor Scores. This figure demonstrates the relation between maternal anxiety self-ratings scores measured through the SCL90-R and infant fine motor scores at 3 months of ages as assessed on the parent-reported EMQ.

The relation between subclinical maternal psychopathology and infants’ early motor development was examined using linear regression. Mother’s depression score, mother’s anxiety score, the child’s age at assessment, and the child’s gender were used predictors for infants’ GM (*M* = −64.49, *SD* = 7.97, *range* −74 to −38), FM (*M* = −64.89, *SD* = 10.32, *range* −78 to −32), and PA (*M* = −41.19, *SD* = 11.66, *range* −63 to 6) skills across three separate models. Overall models were significant in the GM, *F*(4,29) = 5.26, *p* = 0.003, *R^2^_*Adj*_* = 0.34, and FM domains, *F*(4,29) = 4.90, *p* = 0.004, *R^2^_*Adj*_* = 0.32, but not in the PA domain (*p* = 0.635). In the GM domain, only the child’s age (β = 0.57; *p* < 0.001) was a significant predictor of infants’ motor scores. In the FM domain, child age (β = 0.52; *p* = 0.001) was a significant predictor while parent’s anxiety ratings were approaching significance (β = −0.34; *p* = 0.050). Infants of mothers reporting high levels of anxiety showed a trend toward lower FM scores (see Figure 1). Thus, maternal anxiety may have some negative impact on infants’ fine motor skills around 4 months of age.

### Relation Between Maternal Psychopathology and Parent Perceptions

The impact of maternal psychopathology (i.e., depression or anxiety symptoms) on parent’s perceptions about their own child was examined using linear regression Mother’s depression score, mother’s anxiety score, the child’s age at assessment, and child gender were used predictors. The primary outcome measure was a composite score of parent’s perceptions. This composite score was calculated by summing responses across four areas: Positive/Negative perceptions about their role as parent and positive/negative perceptions about their child. Negative perceptions were reverse coded for the composite score (i.e., higher scores indicate the parent endorses overall more positive views). The overall model was significant, *F*(4,27) = 5.16, *p* = 0.003, *R^2^_*Adj*_* = 0.35, with parental depression ratings being the only significant predictor in the model (β = −0.64; *p* < 0.001). This suggest that postnatal maternal depression have a negative impact on parent’s perceptions about their child above and beyond the impact of all other variables in the model. However, it remains unclear what aspects of parent perceptions are most impacted by depression symptoms.

**FIGURE 2:**
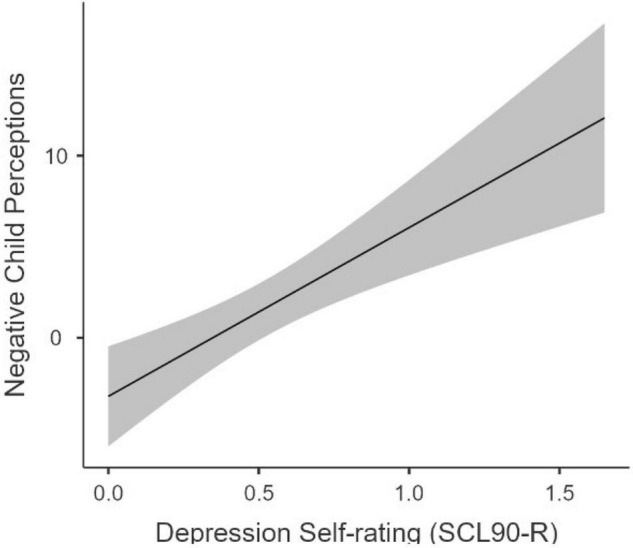
Relation between Maternal Depression and Parents’ Negative Perceptions. This figure demonstrates the relation between maternal depression self-rating scores (SCL90-R) and subsequent parent negative perceptions toward their child (EMQ-X) at around 3 months postpartum.

To determine what kind of parent’s perceptions are most impacted by maternal depression, this analysis was repeated for each of the four parent perception areas separately. Bonferroni correction was applied to these regression results to adjust for multiple comparisons. This analysis failed to reach significance for parent’s positive (*p* = 0.836) or negative perceptions (*p* = 0.256) about their role as parent, and for parent’s positive perceptions about their child (*p* = 0.920). However, the model reached significance for parent’s negative perceptions about their child, *F*(4,28) = 5.14, *p* = 0.012, *R^2^_*Adj*_* = 0.34. Again, parental depression ratings were the only significant predictor in the model (β = 0.74; *p* = 0.004). These results suggest that parents endorsing more symptoms of depression also endorse an overall more negative view toward their child’s development (see Figure 2).

### Fetal Kick Counting and Its Impact on Maternal Psychopathology

The potential impact of completing fetal kick counting during pregnancy on self-endorsed maternal psychopathology symptoms was examined using Analyses of Covariance (ANCOVA) with Fetal Kick Counting (2) and infant Gender (2) as independent variables and child age and parental age as co-variates. Results reveal no significant main effects or interactions regarding depression (*p*s > 0.189) or anxiety ratings (*p*s > 0.182). This suggest that self-endorsed maternal psychopathology symptoms are not affected by engaging in FKC during pregnancy.

**FIGURE 3:**
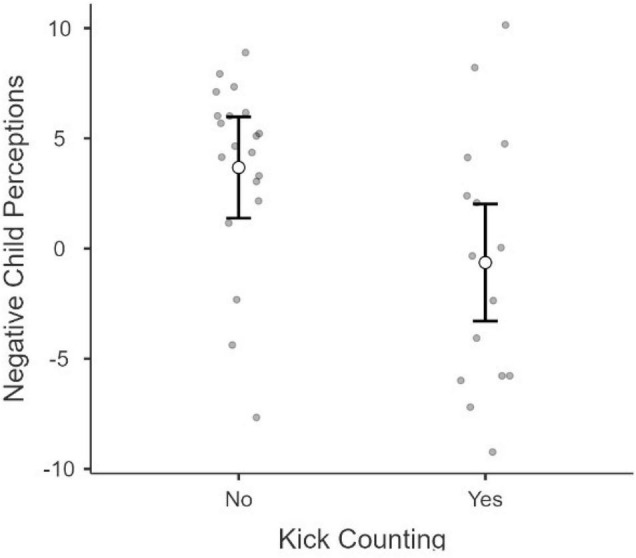
Differences in Parent Negative Perceptions by Fetal Kick Counting Exposure. This figure demonstrates the significant impact seen in the K+ group postnatally as mothers who participated in the brief FKC intervention exhibited a decline in negative perceptions and attitudes toward their infants (EMQ-X) in comparison to the K− group.

To further explore the effect of the FKC activity, we used the same analytic approach to examine if FKC has an impact on parent’s negative perceptions about their child. Our results suggest that mothers self-reporting more depression symptoms seem to also endorse more negative perceptions about their child. While our results provide no evidence for FKC reducing depressive symptoms, FKC may still have a positive impact on mother’s negative perceptions toward their child. Indeed, the model reveals a significant main effect of FKC on parent’s negative perceptions about their child, *F*(1,30) = 6.86, *p* = 0.014 (see Figure 3). There were no other significant effects or interactions (*p*s > 0.195). This suggests that mothers engaging in FCK endorse overall fewer negative perceptions about their child.

### Relation Between Fetal Movements and Infant Motor Development

**FIGURE 4:**
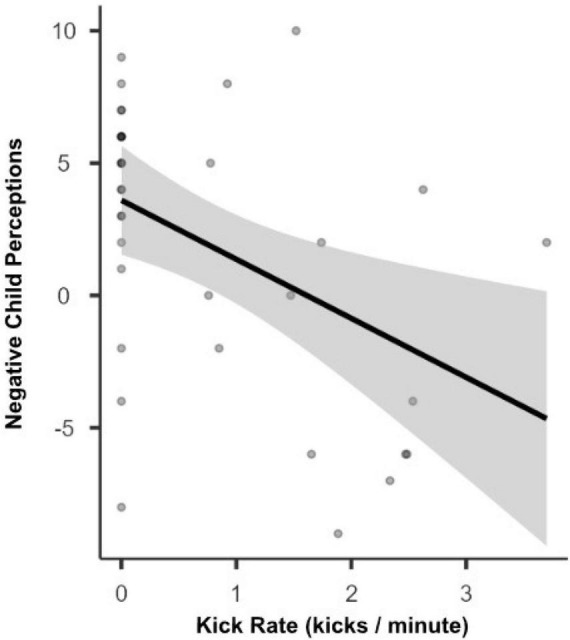
Relation between Fetal Kick Rates and Parent Negative Perceptions. This figure demonstrates the link between *in utero* fetal kick rates (FKC) and negative parent perceptions toward their child (EMQ-X) around 3 months postpartum.

The relation between individual differences in prenatal motor activity (defined as kicks per minute during the FKC activity) and infants’ postnatal motor development as assessed *via* the EMQ was examined using Pearson’s correlations. Further, given the results reported above, we also examined the correlation between prenatal motor activity level and parent’s negative perceptions about their child. Results suggest that average kicks per minute produced during pregnancy were not correlated with infants’ subsequent GM, FM, or PA development (*p*s > 0.435). In contrast, parents; negative perceptions about their child’s development showed a significant negative correlation with the fetus’ average kicks per minute during the FKC activity (*r* = −0.45, *p* = 0.006; see Figure 4). Fetal activity *in utero* did not seem related to postnatal motor development but may impact parents’ subsequent perception of their child.

### Relation Between Fetal Kick Counting and Mother-Child Engagement Quality

Finally, we examined whether engaging in FKC during the third trimester of pregnancy would alter mother-child engagement quality when the child is around 3 months of age. This question was addressed using a 2 (FKC Condition) by 2 (Child Gender) Analysis of Covariance (ANCOVA) controlling for the child’s age at the observation. Results reveal no significant main effects of FKC Condition or Child Gender, and no significant interaction (all *ps* > 0.527). This suggests that mother-child engagement quality, at least as measured in the current study, was not affected by engaging in FKC during pregnancy. However, mother-child dyads in both the K+ (*M* = 3.12, *SD* = 0.86) and K− (*M* = 3.36, *SD* = 0.78) groups scored close to ceiling on the scale. Therefore, it is likely that the scale used here was too narrow in range to detect meaningful differences between the FKC groups.

## Discussion

The present study examined whether subclinical maternal psychopathology (i.e., depression and anxiety) may negatively impact infants’ early motor development. Further, we investigated if fetal kick counting (FKC) may reduce subclinical maternal psychopathology symptoms. And finally, we explored whether engaging in FKC may positively influence subsequent mother-child engagement quality. Results partially confirm our hypotheses. First, we did observe evidence that subclinical maternal psychopathology can have an impact on infants’ early motor skills. Specifically, maternal anxiety symptoms seem to negatively affect infants’ fine motor skills around 3 months of age. Further, we also observed that subclinical maternal psychopathology predicted mothers’ endorsement of negative perceptions toward their child’s development. An increase in mothers’ negative perceptions is one potential route for maternal psychopathology to impact infants’ motor development. Third, while engaging in FKC during pregnancy did not reduce maternal psychopathology symptoms, the FKC activity did seem to reduce mother’s negative perceptions toward their child. Finally, our findings do not suggest an improvement in mother-child engagement after engaging in the FKC activity. Thus, while FKC may have some positive influences, the overall and long-term effect seem relatively small.

Despite not finding a reduction of negative maternal psychopathology from FKC engagement, identifying a relation between fetal movements and negative parental perceptions has important implications and applications. Specifically, depression may manifest in parents as an increase of negative attitudes toward their child’s development. By increasing negative views toward their child, severe and acute depression symptoms may interfere with mother-infant interactions early in life (Tronick and Reck, 2009). The current study examines this issue within the context of subclinical levels of maternal depression and shows that even mild forms of depression may adversely impact aspects of child development. Specifically, our findings suggest that even parents showing only mild depression symptoms seem to endorse more negative perceptions toward their child and their child’s development. This may subsequently impact how the mother interacts with the child. While the current study did not observe any direct evidence this possibility, future research should re-visit this issue. It is possible that the engagement measure used here was not detailed enough to capture the impact of subclinical psychopathology on mother-child engagement. Further, it is also possible that the adverse impact of negative parental perceptions on mother-child engagement require more time to become evident. Longer-term follow-up examinations are needed to address this possibility.

The impact of maternal psychopathology on infant motor development is currently unclear as previous studies have reported inconclusive findings. For example, Piallini et al. (2016) found that children of mothers reporting higher levels of depression show higher motor scores compared to children of mothers experiencing subclinical or no symptoms. In contrast, others report that maternal depression has long-term negative impacts on infant motor development trajectories (Cornish et al., 2005). Our findings provide support for both positions. On the one hand, we report that children of mothers endorsing higher levels of (subclinical) anxiety show overall *lower* fine motor skills at 3 months of age. This suggest that maternal anxiety may have a negative impact on infants’ motor development. On the other hand, we did not observe a relation between subclinical depression symptoms and subsequent infant motor development. Therefore, the relation between subclinical maternal psychopathology and infant motor development does not seem universal but is dependent on both the domain of motor skills assessed (e.g., gross motor vs fine motor) and the type of maternal psychopathology (e.g., depression vs. anxiety). Depression and anxiety symptoms are often comorbid in postpartum depression, and more research is needed to clearly separate their unique or joint impact of on infant motor development.

The potential negative impact of maternal psychopathology highlights the need for intervention in this area. Research regarding the impact of fetal-kick counting is limited, but a few studies report that engaging in FKC may have positive effects. For example, engaging in FKC may increase maternal wellbeing (El-Sayed et al., 2018) and improve general health due to a reduction of anxiety, depression, and insomnia symptoms (Saastad et al., 2012). Unfortunately, the current study did not support the notion that FKC may reduce maternal anxiety or depression symptoms. Nevertheless, mothers in our FKC group endorsed fewer negative perceptions toward their child, suggesting that FKC may have some benefits despite the brief and informal FKC activity applied here. Engaging in FKC for a longer period or using a more structured diary approach may lead to stronger results. Longer-term research is necessary to examine this question.

Another interesting question is whether fetal movements may predict postnatal infant motor development. Past research has discovered links between fetal brain connection patterns and postnatal infant motor development abilities (Thomason et al., 2018). In addition, prenatal fetal activity levels have been shown to correlate with postnatal infant activity levels (DiPietro et al., 2002). However, our findings did not observe a link between prenatal motor activity and postnatal infant motor development trajectories. This may be due to using maternal self-report to assess fetal movement activity. More accurate and objective measures of fetal movement activity (e.g., ultrasound) should re-visit this idea to determine if fetal movement activity can predict postnatal motor skill emergence.

The current study also examined whether engaging in FKC during pregnancy might alter mother-child engagement quality following birth. One could speculate that engaging in FKC may encourage bonding between mother and child prior to birth and enhance the subsequent quality of mother-child interactions. Such a relation between FKC and mother-child interactions could act as a potential mechanism explaining some of the other findings reported here. Specifically, improved mother-child engagement may encourage infant motor skills and decrease the likelihood of maternal psychopathology symptoms. Our findings do not support that FKC influences subsequent mother-infant engagement. However, it is possible that the engagement scale used here (see Table 2) could not adequately capture nuances in mother-infant engagement.

Finally, it is important to note that the current study only focused on the relation between the *mother* and child. Our FKC activity had to be completed by the mother, and our postnatal psychopathology assessment focused on the mother only. However, it is possible that FKC could also be performed by fathers/partners, and the joint activity with the pregnant mother may be more effective than FKC by the mother alone. Further, fathers also experience postpartum depression and are influenced by similar psychosocial factors as mothers (Escribà-Agüir and Artazcoz, 2011), although fewer cases are diagnosed due to cultural and gender expectations (Hops, 1995). Fathers experiencing postpartum depression are more likely to endure a constant state of depression, whereas mothers are likely to decrease in symptomatology over the course of the first year postpartum (Escribà-Agüir and Artazcoz, 2011). Therefore, postpartum depression is an important issue with fathers as well. Fathers are a critical factor predicting early child development and the interactions between father and child are likely to have long-term implications for child development. Therefore, examining the benefits of FKC with fathers presents an interesting next step for this line of research.

### Limitations

Some limitations need to be considered when interpreting our results. First, the sample size was relatively small and lacked diversity. This limits the generalizability of our results. Further, the engagement coding scheme used here may not fully encapsulate the complex dynamics of mother-infant engagement. The measure used here primarily assesses the reciprocal nature of mother-infant interactions, although research on depressed and anxious mothers shows them to also exhibit negative characteristics such as hostility and intrusiveness (Wang and Dix, 2013). These behaviors were not quantified in the current study. Finally, mother-infant dyads were not randomly assigned to group membership. Instead, assignment into groups was based on the time of recruitment into the study. However, all families agree to participate in a larger longitudinal study where most study related observations only begin after the child is around 3-months of age. Together with the lack of demographic differences (see Table 1), there is little reason to suggest that families self-selected into the FKC group on purpose. Nevertheless, we acknowledge that the lack of random assignment needs to be considered when interpreting our results. Finally, depression may significantly affect maternal behavior and compliance with prenatal routines. None of the mothers enrolled in the current study presented with clinical levels of depression or anxiety on our self-report screening measure. Self-report may include under (or over) reporting of symptoms and could have impacted our results.

### Future Directions

The results reported here show promise for the use of FKC as a simple and cost-efficient intervention. Future research should further explore this possibility and improve upon the FKC approach used here. The current study did not combine the FKC activity with any form of parental education or even provide any feedback about the child’s behavior. Others used a similar approach by explicitly incorporated fetal kick counting informational sessions for mothers to learn more about self-monitoring fetal kicks and to potentially stimulate fetal movements (El-Sayed et al., 2018). This information may increase mothers’ investment and enjoyment. Future studies should combine fetal kick counting with information about fetal development. A mobile app called “Mamma Mia” used a similar approach to combat postpartum depression using a combination of positive and metacognitive psychology in addition to couples therapy (Haga et al., 2013). In combination with a parent-child bonding activity such as FKC, effectiveness of this approach may be increased. However, potential risks such as the FKC activity inadvertently inducing anxiety also need to be considered and this potential side effect needs to be examined before broadly recommending FKC.

## Conclusion

The current findings advance our understanding of the potential benefits of engaging in prenatal fetal kick counting for postnatal infant motor development and long-term mother-infant interactions. Results suggest that mothers experiencing subclinical levels of depression may subsequently exhibit more negative perceptions toward their child. Furthermore, the presence of subclinical maternal anxiety seems to have some influence on infants’ fine motor skills development. Specifically, engaging in fetal kick counting during the third trimester of pregnancy may reduce parents’ negative perceptions toward their child. Given the low cost and ease of implementation, our preliminary study suggests that fetal kick counting is a worthwhile activity that should be examined further in future research.

## Data Availability Statement

The raw data supporting the conclusions of this article will be made available by the authors, without undue reservation.

## Ethics Statement

The studies involving human participants were reviewed and approved by University of Pittsburgh IRB. Written informed consent to participate in this study was provided by the participants’ legal guardian/next of kin.

## Author Contributions

KL and BO were completed the data analysis, worked on subsequent revisions of the manuscript, and involved in the conceptualization and design of the study. KL was involved in participant recruitment and data collection. BO completed the first draft of the manuscript, data coding, and cleaning. Both authors contributed to the article and approved the submitted version.

## Conflict of Interest

The authors declare that the research was conducted in the absence of any commercial or financial relationships that could be construed as a potential conflict of interest.

## Publisher’s Note

All claims expressed in this article are solely those of the authors and do not necessarily represent those of their affiliated organizations, or those of the publisher, the editors and the reviewers. Any product that may be evaluated in this article, or claim that may be made by its manufacturer, is not guaranteed or endorsed by the publisher.

## References

[B1] Ainsworth M. S. (1979). Infant–mother attachment. *Am. Psychol.* 34 932–937. 10.1037/0003-066X.34.10.932 517843

[B2] Barker E. D. Jaffee S. R. Uher R. Maughan B. (2011). The contribution of prenatal and postnatal maternal anxiety and depression to child maladjustment. *Depress. Anxiety* 28 696–702. 10.1002/da.20856 21769997

[B3] Bauman B. L. Ko J. Y. Cox S. D’Angelo D. V. Warner L. Folger S. (2020). Vital signs: postpartum depressive symptoms and provider discussions about perinatal depression—United States, 2018. *Morbid. Mortal. Wkly. Rep.* 69:575. 10.15585/mmwr.mm6919a2 32407302 PMC7238954

[B4] Bernard-Bonnin A.-C. Society C. P. Health M. Committee D. D. (2004). Maternal depression and child development. *Paediatr. Child Health* 9 575–583. 10.1093/pch/9.8.575 19680490 PMC2724169

[B5] Brookman R. Kalashnikova M. Conti J. Xu Rattanasone N. Grant K.-A. Demuth K. (2020). Depression and anxiety in the postnatal period: an examination of infants’ home language environment, vocalizations, and expressive language abilities. *Child Dev.* 91 e1211–e1230. 10.1111/cdev.13421 32745250

[B6] Buist A. (2002). Mental health in pregnancy: the sleeping giant. *Austr. Psychiatry* 10 1–4.

[B7] Carolan M. (2005). “Doing it properly”: the experience of first mothering over 35 years. *Health Care Women Int.* 26 764–787. 10.1080/07399330500230987 16214793

[B8] Castelli R. D. Quevedo Lde Á Coelho F. M. Lopez M. A. da Silva R. A. Böhm D. M. (2015). Cognitive and language performance in children is associated with maternal social anxiety disorder: a study of young mothers in southern Brazil. *Early Hum. Dev.* 91 707–711. 10.1016/j.earlhumdev.2015.10.002 26544906

[B9] Chess S. Thomas A. (1982). Infant bonding: mystique and reality. *Am. J. Orthopsychiatr.* 52:213. 10.1111/j.1939-0025.1982.tb02683.x 7081393

[B10] Cicchetti D. Rogosch F. A. Toth S. L. (2000). The efficacy of toddler-parent psychotherapy for fostering cognitive development in offspring of depressed mothers. *J. Abnormal Child Psychol.* 28 135–148. 10.1023/a:1005118713814 10834766

[B11] Cornish A. M. McMahon C. A. Ungerer J. A. Barnett B. Kowalenko N. Tennant C. (2005). Postnatal depression and infant cognitive and motor development in the second postnatal year: the impact of depression chronicity and infant gender. *Infant Behav. Dev.* 28 407–417. 10.1016/j.infbeh.2005.03.004

[B12] Cummings E. M. Davies P. T. (1994). Maternal depression and child development. *J. Child Psychol. Psychiatry* 35 73–122. 8163630 10.1111/j.1469-7610.1994.tb01133.x

[B13] Davey H. L. Tough S. C. Adair C. E. Benzies K. M. (2011). Risk factors for sub-clinical and major postpartum depression among a community cohort of Canadian women. *Mater. Child Health J.* 15 866–875. 10.1007/s10995-008-0314-8 18256913

[B14] Delaram M. Jafar-Zadeh L. Shams S. (2017). Fetal movement counting and maternal depression: a randomized, controlled trial. *Zahedan J. Res. Med. Sci.* 19:e5680.

[B15] Derogatis L. R. (1983). *SCL-90–R: Manual II.* Towson, MD: Clinical Psychometric Research.

[B16] DiPietro J. A. Bornstein M. H. Costigan K. A. Pressman E. K. Hahn C. S. Painter K. (2002). What does fetal movement predict about behavior during the first two years of life? *Dev. Psychobiol.* 40 358–371. 10.1002/dev.10025 12115294

[B17] El-Sayed H. El-Sayed M. Hassan S. I. Hakeem S. A. Aboud H. Ibrahim A. (2018). Effect of women self monitoring of fetal kicks on enhancing their general health status. *Am. J. Nurs. Res.* 6 117–124.

[B18] Escribà-Agüir V. Artazcoz L. (2011). Gender differences in postpartum depression: a longitudinal cohort study. *J. Epidemiol. Commun. Health* 65 320–326. 10.1136/jech.2008.085894 20515899 PMC3069755

[B19] Figueiredo B. Canário C. Field T. (2014). Breastfeeding is negatively affected by prenatal depression and reduces postpartum depression. *Psychol. Med.* 44 927–936. 10.1017/S0033291713001530 23822932

[B20] Fogel J. Eaton W. W. Ford D. E. (2006). Minor depression as a predictor of the first onset of major depressive disorder over a 15-year follow-up. *Acta Psychiatr. Scand.* 113 36–43. 10.1111/j.1600-0447.2005.00654.x 16390367

[B21] Folio M. Fewell R. (2000). *Peabody Developmental Motor Scales.* Austin, TX: Pro-Ed. Inc.

[B22] Fredriksen E. von Soest T. Smith L. Moe V. (2019). Parenting stress plays a mediating role in the prediction of early child development from both parents’ perinatal depressive symptoms. *J. Abnormal Child Psychol.* 47 149–164. 10.1007/s10802-018-0428-4 29623542

[B23] Haga S. M. Drozd F. Brendryen H. Slinning K. (2013). Mamma mia: a feasibility study of a web-based intervention to reduce the risk of postpartum depression and enhance subjective well-being. *JMIR Res. Protoc.* 2:e29. 10.2196/resprot.2659 23939459 PMC3742405

[B24] Hops H. (1995). Age-and gender-specific effects of parental depression: a commentary. *Dev. Psychol.* 31:428. 10.1037/0012-1649.31.3.428

[B25] Ibanez G. Bernard J. Y. Rondet C. Peyre H. Forhan A. Kaminski M. (2015). Effects of antenatal maternal depression and anxiety on children’s early cognitive development: a prospective cohort study. *PLoS One* 10:e0135849. 10.1371/journal.pone.0135849 26317609 PMC4552796

[B26] Katherine Weinberg M. Olson K. L. Beeghly M. Tronick E. Z. (2006). Making up is hard to do, especially for mothers with high levels of depressive symptoms and their infant sons. *J. Child Psychol. Psychiatry* 47 670–683. 10.1111/j.1469-7610.2005.01545.x 16790002

[B27] Lewin A. Mitchell S. J. Ronzio C. R. (2013). Developmental differences in parenting behavior: comparing adolescent, emerging adult, and adult mothers. *Merrill Palmer Q.* 59 23–49. 10.1007/s00737-021-01116-5 33742282

[B28] Libertus K. Landa R. J. (2013). The early motor questionnaire (EMQ): a parental report measure of early motor development. *Infant Behav. Dev.* 36 833–842. 10.1016/j.infbeh.2013.09.007 24140841 PMC3858411

[B29] Libertus K. Violi D. A. (2016). Sit to talk: relation between motor skills and language development in infancy. *Front. Psychol.* 7:475. 10.3389/fpsyg.2016.00475 27065934 PMC4815289

[B30] Lindgren K. (2001). Relationships among maternal–fetal attachment, prenatal depression, and health practices in pregnancy. *Res. Nurs. Health* 24 203–217. 10.1002/nur.1023 11526619

[B31] Liston R. M. Bloom K. Zimmer P. (1994). The psychological effects of counting fetal movements. *Birth* 21 135–140. 10.1111/j.1523-536x.1994.tb00512.x 7857455

[B32] Lobb M. Beazley J. Haddad N. (1985). A controlled study of daily fetal movement counts in the prevention of stillbirths. *J. Obstetr. Gynaecol.* 6 87–91. 10.3109/01443618509079151

[B33] Masten A. S. Cicchetti D. (2010). Developmental cascades. *Dev. Psychopathol.* 22 491–495. 10.1017/S0954579410000222 20576173

[B34] Matsumoto K. Tsuchiya K. J. Itoh H. Kanayama N. Suda S. Matsuzaki H. (2011). Age-specific 3-month cumulative incidence of postpartum depression: the Hamamatsu birth Cohort (HBC) study. *J. Affect. Disord.* 133 607–610. 10.1016/j.jad.2011.04.024 21601291

[B35] Milgrom J. Gemmill A. W. Bilszta J. L. Hayes B. Barnett B. Brooks J. (2008). Antenatal risk factors for postnatal depression: a large prospective study. *J. Affect. Disord.* 108 147–157. 10.1016/j.jad.2007.10.014 18067974

[B36] Milgrom J. Westley D. T. Gemmill A. W. (2004). The mediating role of maternal responsiveness in some longer term effects of postnatal depression on infant development. *Infant Behav. Dev.* 27 443–454. 10.1016/j.infbeh.2004.03.003

[B37] Mullen E. M. (1995). *Mullen Scales of Early Learning.* AGS. Circle Pines, MN: American Guidance Service Inc.

[B38] Müller D. Teismann T. Havemann B. Michalak J. Seehagen S. (2013). Ruminative thinking as a predictor of perceived postpartum mother–infant bonding. *Cogn. Ther. Res.* 37 89–96.

[B39] Murray L. Kempton C. Woolgar M. Hooper R. (1993). Depressed mothers’ speech to their infants and its relation to infant gender and cognitive development. *J. Child Psychol. Psychiatry* 34 1083–1101. 10.1111/j.1469-7610.1993.tb01775.x 8245134

[B40] O’Connor T. G. Heron J. Golding J. Beveridge M. Glover V. (2002). Maternal antenatal anxiety and children’s behavioural/emotional problems at 4 years: report from the avon longitudinal study of parents and children. *Br. J. Psychiatry* 180 502–508. 10.1192/bjp.180.6.502 12042228

[B41] Paulson J. F. Keefe H. A. Leiferman J. A. (2009). Early parental depression and child language development. *J. Child Psychol. Psychiatry* 50 254–262. 10.1111/j.1469-7610.2008.01973.x 19175819

[B42] Piallini G. Brunoro S. Fenocchio C. Marini C. Simonelli A. Biancotto M. (2016). How do maternal subclinical symptoms influence infant motor development during the first year of life? *Front. Psychol.* 7:1685. 10.3389/fpsyg.2016.01685 27847489 PMC5088190

[B43] Reck C. Struben K. Backenstrass M. Stefenelli U. Reinig K. Fuchs T. (2008). Prevalence, onset and comorbidity of postpartum anxiety and depressive disorders. *Acta Psychiatr. Scand.* 118 459–468. 10.1111/j.1600-0447.2008.01264.x 18840256

[B44] Rezaie-Keikhaie K. Arbabshastan M. E. Rafiemanesh H. Amirshahi M. Ostadkelayeh S. M. Arbabisarjou A. (2020). Systematic review and meta-analysis of the prevalence of the maternity blues in the postpartum period. *J. Obst. Gynecol. Neonatal Nurs.* 49 127–136. 10.1016/j.jogn.2020.01.001 32035973

[B45] Robertson E. Grace S. Wallington T. Stewart D. E. (2004). Antenatal risk factors for postpartum depression: a synthesis of recent literature. *Gen. Hosp. Psychiatry* 26 289–295. 10.1016/j.genhosppsych.2004.02.006 15234824

[B46] Saastad E. Israel P. Ahlborg T. Gunnes N. Frøen J. F. (2011). Fetal movement counting—effects on maternal-fetal attachment: a multicenter randomized controlled trial. *Birth* 38 282–293. 10.1111/j.1523-536X.2011.00489.x 22112328

[B47] Saastad E. Winje B. A. Israel P. Frøen J. F. (2012). Fetal Movement counting—maternal concern and experiences: a multicenter, randomized, controlled trial. *Birth* 39 10–20. 10.1111/j.1523-536X.2011.00508.x 22369601

[B48] Schmidt D. Seehagen S. Vocks S. Schneider S. Teismann T. (2016). Predictive importance of antenatal depressive rumination and worrying for maternal–Foetal attachment and maternal well-being. *Cogn. Ther. Res.* 40 565–576.

[B49] Thomason M. E. Hect J. Waller R. Manning J. H. Stacks A. M. Beeghly M. (2018). Prenatal neural origins of infant motor development: associations between fetal brain and infant motor development. *Dev. Psychopathol.* 30:763. 10.1017/S095457941800072X 30068433 PMC6261435

[B50] Tronick E. Reck C. (2009). Infants of depressed mothers. *Harv. Rev. Psychiatry* 17 147–156. 10.1080/10673220902899714 19373622

[B51] Van Egeren L. A. Barratt M. S. Roach M. A. (2001). Mother–infant responsiveness: timing, mutual regulation, and interactional context. *Dev. Psychol.* 37:684. 10.1037//0012-1649.37.5.684 11552763

[B52] Wang Y. Dix T. (2013). Patterns of depressive parenting: why they occur and their role in early developmental risk. *J. Family Psychol.* 27 884. 10.1037/a0034829 24294931

