# Supplementary material for: Are There Postnatal Benefits to Prenatal Kick Counting? A Quasi-Experimental Longitudinal Study
Source: Front Psychol. 2022 Jan 26;13:712562. doi: 10.3389/fpsyg.2022.712562 (PMC8825801; doi:10.3389/fpsyg.2022.712562)
Supplement: Supplementary file 1 [file Table_1.docx]

**Appendix 1**

**Early Motor Questionnaire – Parent Perceptions Extension (20 items)**

*The following section is about your experiences and attitudes towards parenting and your child.*

**How do you feel about parenting in general?**

|  | *Strongly disagree* | *Somewhat disagree* | *Neither agree nor disagree* | *Somewhat agree* | *Strongly agree* |
| --- | --- | --- | --- | --- | --- |
| 1) I enjoy being a parent |  |  |  |  |  |
| 2) Parenting is easy for me |  |  |  |  |  |
| 3) I feel good about my parenting skills |  |  |  |  |  |
| 4) I would like to spend more time with my child |  |  |  |  |  |
| 5) Parenting has enriched my life |  |  |  |  |  |

**Compared to other children you know, your child**

|  | *Strongly disagree* | *Somewhat disagree* | *Neither agree nor disagree* | *Somewhat agree* | *Strongly agree* |
| --- | --- | --- | --- | --- | --- |
| 6) is easier going than most children |  |  |  |  |  |
| 7) smiles a lot |  |  |  |  |  |
| 8) is smarter than most children |  |  |  |  |  |
| 9) is very social and engaging |  |  |  |  |  |
| 10) communicates with you clearly |  |  |  |  |  |

**Being a parent means**

|  | *Strongly disagree* | *Somewhat disagree* | *Neither agree nor disagree* | *Somewhat agree* | *Strongly agree* |
| --- | --- | --- | --- | --- | --- |
| 11) having less time for yourself |  |  |  |  |  |
| 12) having more problems than before |  |  |  |  |  |
| 13) feeling sad more often than before |  |  |  |  |  |
| 14) arguing more with your partner |  |  |  |  |  |
| 15) being burdened with responsibilities |  |  |  |  |  |

**On a bad day, your child**

|  | *Strongly disagree* | *Somewhat disagree* | *Neither agree nor disagree* | *Somewhat agree* | *Strongly agree* |
| --- | --- | --- | --- | --- | --- |
| 16) may scream loudly |  |  |  |  |  |
| 17) refuses to be comforted by you |  |  |  |  |  |
| 18) does not participate in routines |  |  |  |  |  |
| 19) is less predictable than usual |  |  |  |  |  |
| 20) is hard to soothe |  |  |  |  |  |
